# Differential Effects of Paraquat, Rotenone, and MPTP on Cellular Bioenergetics of Undifferentiated and Differentiated Human Neuroblastoma Cells

**DOI:** 10.3390/brainsci13121717

**Published:** 2023-12-14

**Authors:** Ekramy Elmorsy, Ayat Al-Ghafari, Huda Al Doghaither, Sara Hashish, Mohamed Salama, Anusha W. Mudyanselage, Lipta James, Wayne G. Carter

**Affiliations:** 1 Department of Forensic Medicine and Clinical Toxicology, Faculty of Medicine, Mansoura University, Mansoura 35516, Egypt; 2 Pathology Department, Faculty of Medicine, Northern Border University, Arar 91431, Saudi Arabia; 3 Department of Biochemistry, Faculty of Science, King Abdulaziz University, Jeddah 21589, Saudi Arabia; abalghafari@kau.edu.sa (A.A.-G.); haldoghaither@kau.edu.sa (H.A.D.); 4 Experimental Biochemistry Unit, King Fahd Medical Research Center, King Abdulaziz University, Jeddah 21589, Saudi Arabia; 5 Institute of Global Health and Human Ecology, The American University in Cairo (AUC), Cairo 11385, Egypt; sarahashish@aucegypt.edu (S.H.); mohamed-salama@aucegypt.edu (M.S.); 6 Clinical Toxicology Research Group, School of Medicine, University of Nottingham, Royal Derby Hospital Centre, Derby DE22 3DT, UK; wijesekara@agri.sab.ac.lk (A.W.M.); liptajames25@gmail.com (L.J.); 7 Faculty of Agricultural Sciences, Sabaragamuwa University of Sri Lanka, Belihuloya 70140, Sri Lanka

**Keywords:** 1-methyl-4-phenyl-1,2,3,6-tetrahydropyridine (MPTP), mitochondrial damage, neurotoxicity, oxidative stress, paraquat, pesticides, rotenone

## Abstract

Paraquat (PQ), rotenone (RO), and 1-methyl-4-phenyl-1,2,3,6-tetrahydropyridine (MPTP) are neurotoxicants that can damage human health. Exposure to these neurotoxicants has been linked to neurodegeneration, particularly Parkinson’s disease. However, their mechanisms of action have not been fully elucidated, nor has the relative vulnerability of neuronal subtypes to their exposures. To address this, the current study investigated the cytotoxic effects of PQ, RO, and MPTP and their relative effects on cellular bioenergetics and oxidative stress on undifferentiated human neuroblastoma (SH-SY5Y) cells and those differentiated to dopaminergic (DA) or cholinergic (CH) phenotypes. The tested neurotoxicants were all cytotoxic to the three cell phenotypes that correlated with both concentration and exposure duration. At half-maximal effective concentrations (EC_50_s), there were significant reductions in cellular ATP levels and reduced activity of the mitochondrial complexes I and III, with a parallel increase in lactate production. PQ at 10 µM significantly decreased ATP production and mitochondrial complex III activity only in DA cells. RO was the most potent inhibitor of mitochondrial complex 1 and did not inhibit mitochondrial complex III even at concentrations that induced a 50% loss of cell viability. MPTP was the most potent toxicant in undifferentiated cells. All neurotoxicants significantly increased reactive oxygen species, lipid peroxidation, and nuclear expression of Nrf2, with a corresponding inhibition of the antioxidant enzymes catalase and superoxide dismutase. At a 10 µM exposure to PQ or RO, oxidative stress biomarkers were significant in DA cells. Collectively, this study underscores the importance of mitochondrial dysfunction and oxidative stress in PQ, RO, and MPTP-induced cytotoxicity and that neuronal phenotypes display differential vulnerability to these neurotoxicants.

## 1. Introduction

Parkinson’s disease (PD) is a progressive neurodegenerative disease typified by motor and non-motor symptoms and affects more than 6.2 million people globally [1]. Monogenic inheritance only accounts for 5–10% of PD cases, whereas the etiology for the remaining 90% of patients arises from a complex interplay of genetic, environmental, and lifestyle influences [2].

1-methyl-4-phenylpyridinium (MPP^+^), an impurity in the preparation of heroin, was found to induce neurodegeneration and parkinsonism in drug abusers [3]. MPP^+^ has been marketed as a pesticide under the trade name Cyperquat [4], and since it shows structural similarity to other pesticides, including paraquat (PQ), this triggered research that considered the potential association between pesticides and PD. Subsequently, three meta-analyses of epidemiological studies have concluded that pesticide exposure and related factors such as rural living, farming, and well-water drinking have an increased relative risk of PD [5,6,7,8].

PQ (N,N′-dimethyl-4-4-4′-bipyridinium) is a divalent cationic herbicide with a chemical structure similar to MPP^+^ [9]. The post-mortem examination of eight patients who died from PQ poisoning revealed diffuse edema as well as astrocyte and microglial activation in their brains [10]. Although structurally similar to MPP^+^, PQ adopts a different mechanism to cause neurotoxicity. It causes cellular oxidative stress by generating reactive oxygen species (ROS) [11]. Once in the brain, PQ can enter central nervous system (CNS) cells in a sodium-dependent fashion, where it acts as a redox cycling compound and damages mitochondria [12,13]. PQ triggers lipid peroxidation, and the levels of endogenous antioxidants such as glutathione are depleted, contributing to cellular redox stress and the induction of mitochondrial apoptosis [12,13].

Rotenone (RO) is a natural plant pesticide from the Leguminosae family [14]. RO is highly lipophilic and readily crosses the blood-brain barrier (BBB). It can inhibit complex I of the electron transport chain, which results in reduced ATP production and the generation of ROS and thereby acts as a redox stressor [15,16]. Additionally, RO inhibits the activity of the proteasome, either through complex I or complex III inhibition or from increased nitric oxide production [17], and induces microtubule destabilization [18].

MPTP (1-methyl-4-phenyl-1,2,3,6-tetrahydropyridine) induces PD-like conditions when administered to animals. Like RO, MPTP is highly lipophilic and ably crosses the BBB, where it is metabolized in astrocytes by monoamine oxidase-B into MPP^+^, a more potent neurotoxicant [19]. MPP^+^ is released from astrocytes by the organic cation transporter-3 and is moved into the extracellular space [20], where it can be taken up by dopamine transporters into dopaminergic neurons. MPP^+^ acts as a mitochondrial complex I inhibitor, with associated ATP depletion and oxidative stress [21]. However, mitochondrial complex 1 inhibition may not be required for MPP^+^-induced death of dopaminergic neurons [22].

With the recent advancement in mitochondrial function studies, including both its dynamic interactions and role in cellular homoeostasis, a growing body of evidence supports a major role of mitochondrial dysfunction in the development of sporadic PD. In this context, respiratory chain impairment represents a key feature in sporadic PD patients, and there is growing support that links PD-related genes with mitochondrial dysfunction [2].

Although other studies have considered the neurotoxicity of PQ, RO, and MPTP individually, a direct comparison of their neurotoxic effects on undifferentiated human neuroblastoma cells (SH-SY5Y) and those differentiated into dopaminergic (DA) and cholinergic (CH) cells has not previously been undertaken. Furthermore, this study specifically considered the relative phenotype vulnerability to these neurotoxicants in terms of their potential for detrimental impacts on cellular bioenergetics and the induction of redox stress.

## 2. Materials and Methods

### 2.1. Chemicals and Reagents

Unless otherwise specified, all chemicals, including PQ dichloride, RO, and MPTP, were purchased from Sigma-Aldrich (Poole, UK). Stock solutions for PQ dichloride and MPTP were prepared in culture media, while RO was first dissolved in ethanol, then added to the media to a final ethanol concentration of 200 µM. The prepared media was used for control cells in RO experiments. For ATP assays, a commercial kit was obtained from Abcam (Cambridge, UK), and a commercial lactate assay kit was purchased from Biovision (Mountain View, CA, USA).

Regarding the buffers for the mitochondrial complex assays, for each 10 mL of complex I assay buffer, the following chemicals were used: 5 mL of potassium phosphate buffer (50 mM pH 7.6), 2 mL of dichloroindophenol sodium salt hydrate (DCIP) stock (0.6 mM), 140 µL of decylubiquinone (DUB) (5 mM), and 10 µL of antimycin A (1 mM). The volume was adjusted to 10 mL with distilled water, and then fatty acid-free bovine serum albumin (BSA) (35 mg) was added. For each 10 mL of mitochondrial complex III buffer, the following chemicals were used: 500 μL of potassium phosphate buffer (0.5 M, pH 7.5), 750 μL of oxidized cytochrome c, 400 μL of sodium azide (10 mM), 20 μL of ethylenediaminetetraacetic acid (EDTA) (5 mM, pH 7.5), and 10 μL of Tween-20 (2.5%) (*v*/*v*). The final volume was adjusted to 10 mL with distilled water.

### 2.2. Cell Culture

SH-SY5Y cells were purchased from the European Collection of Authenticated Cell Culture (ECACC) (ECACC-94030304). Cells were used between passages 13–14 and were grown in a culture medium composed of 43.5% Eagle’s Minimum Essential Medium (EMEM) (M4655, Sigma-Aldrich, Poole, UK) supplemented with 43.5% Ham’s F12 nut mix (217665-029, Gibco, Waltham, MA, USA), 10% heat-inactivated Fetal Bovine Serum (FBS) (F9665, Sigma-Aldrich, Poole, UK), 1% MEM Non-Essential Amino Acid Solution (NEAA) (RNBF3937, Sigma, Poole, UK), 2 mM glutamine, and 1% penicillin–streptomycin solution containing 10,000 IU penicillium and 10 mg/mL streptomycin (p/s) (P4333, Sigma-Aldrich, Poole, UK) in 25 or 75 cm^2^ flasks (Thermofisher scientific, Rochester, UK) at 37 °C with an atmosphere of 5% CO_2_ and 95% humidity. Cells were checked daily until they attained 80% confluence, after which the culture medium was changed every other day.

### 2.3. SH-SY5Y Cell Differentiation

SH-SY5Y cells were seeded in either poly-D-Lysine hydrobromide (5 mg/mL) (P6407, Sigma-Aldrich, Poole, UK)-coated 25 cm^2^ flasks (T25, 130189, Thermofisher Scientific, Rochester, UK) or in 96-well microtiter plates (6005649, Perkin Elmer, Groningen, The Netherlands) with 10% FBS media and, after settling, grown until 60% confluency was attained.

#### 2.3.1. Differentiation to Produce Cells of a Cholinergic Phenotype

The day after the cells reached 60% confluence, they were treated with differentiation media (10 µM *all-trans* retinoic acid (RA)) (R2625, Sigma-Aldrich, Poole, UK) in high serum SH-SY5Y medium (2.5% FBS) for 6 days, with medium changes on days three and five. On day seven, the media were removed, and the cells were trypsinized, split at a 1:1 ratio, and incubated in fresh differentiation media. On day eight, the media were aspirated, and the cells were treated with 10 µM RA in low serum SH-SY5Y medium (1% FBS) for 48 h. On day ten, cells were trypsinized, split at a 1:1 ratio, and incubated in the same differentiation media for 24 h. The media were aspirated, and the cells were treated with medium containing 50 ng/mL brain-derived neurotrophic factor (BDNF) (B3795, Sigma-Aldrich, Poole, UK) and low serum media containing RA for a further 2 days, after which the cells displayed a fully differentiated morphology [23] as described in a previous publication [24] (Supplementary Figure S1). Plated cells were protected from light throughout the differentiation period to prevent the disassociation of RA.

#### 2.3.2. Differentiation to Produce Cells of a Dopaminergic Phenotype

Cells were grown in SH-SY5Y media containing 10 µM RA and stored in the dark for 3 days, and then the media were removed and replaced with fresh RA media for a further 3 days. The media were replaced and cells were grown in media containing 80 nM 12 *O*-tetradecanoyl-phorbol-13-acetate (TPA) for 3 days of differentiation, then the media were aspirated and replaced with fresh TPA media for another 3 days of differentiation, similar to the methods described elsewhere [25].

### 2.4. Cytotoxicity and Cell Viability Assessment Using an MTT Assay

The cytotoxic effect of PQ, RO, and MPTP on undifferentiated SH-SY5Y cells and CH and DA cells was evaluated using a 3-(4,5-dimethylthiazol,2-yl)-2,5-diphenyl tetrazolium bromide (MTT) assay. The MTT assay was employed as a general cell viability assay reliant on the ability of healthy cells to reduce the MTT substrate to insoluble formazan primarily through the activity of NADPH oxidoreductase enzymes [26]. Assay optimization was undertaken to ensure that the MTT signal was linear over the course of the assay (up to 48 h) and with the number of cells seeded (refer to Supplementary Figure S2) and conducted according to a previous publication [27]. Briefly, SH-SY5Y cells were subcultured in a 96-well plate and either grown undifferentiated (seeded at 1 × 10^4^ cells/well) or differentiated into either CH or DA neuronal phenotypes. SH-SY5Y cells were treated with PQ, RO, or MPTP at concentrations of 0.1, 1, 10, 100, or 1000 μM for 3, 6, 12, 24, and 48 h. A total of 10 µL of MTT reagent was added to each well for undifferentiated SH-SY5Y cells, and 15 µL was added to the wells containing differentiated CH or DA cells to ensure the MTT signal was sufficient for the experimental time course. Cells were incubated for 2 h, after which 100 µL of solubilizing reagent was added to each well. After 1 h of incubation, the absorbance at 590 nm was read using a Dyne MRX micro-plate reader (Dyne technologies, Chantilly, VA, USA) after shaking at medium speed for 30 s. MTT absorbance values were expressed as a percentage of the corresponding value of controls (assumed to be 100% viability) after subtracting values from blank wells. Each treatment was performed in triplicate per experimental plate, and each experiment was performed at least three times.

### 2.5. Bioenergetics Assays

#### 2.5.1. Intracellular ATP Measurements

Cells were seeded in 96-well plates and treated with PQ, RO, or MPTP at their estimated MTT EC_50_ concentrations (15, 90, and 150 µM, respectively). Cells were also treated with the tested neurotoxicant at a concentration of 10 µM to compare their effects on ATP levels at a lower fixed concentration. After 24 h, the levels of intracellular ATP were measured using a commercial kit according to the manufacturer’s protocol (Abcam, Cambridge, UK). Briefly, 50 µL of cell lysis buffer containing an ATPase inhibitor was added to each well, and the plate was shaken for 5 min. Then, 50 µL of reconstituted substrate was added in each well and shaken for a further 5 min, and then kept in the dark for 10 min before measuring the signal generation using a luminometer ‘TopCount’ (Perkin Elmer, Ueberlingen, Germany). The medium blank values were subtracted from the readings of all wells, and the readings were normalized to the protein content per well. Experiments were performed in triplicate, with a minimum of three experiments per treatment concentration.

#### 2.5.2. Mitochondrial Complexes I and III Activity Measurements

To assess the impact of the neurotoxicants on mitochondrial functioning, mitochondrial activity assay measurements were undertaken. Activity assays for mitochondrial complexes I and III were performed after treatment with PQ, RO, or MPTP at their estimated MTT EC_50_ concentrations (15, 90, and 150 µM, respectively) and at a lower concentration of 10 µM for 24 h.

#### 2.5.3. Mitochondrial Complex I Assay

For the complex I assay, mitochondrial-enriched fractions were prepared based on the protocol described in Elmorsy et al. [28]. The mitochondrial solution was subjected to three freeze–thaw cycles in liquid nitrogen to disrupt the mitochondrial membranes. For assays, 200 µL of the assay buffer was added per well in 96-well plates. Then, 10 µL of 1 mM RO was added to 3 wells. After that, 3 µg of protein from the isolated freeze–thawed mitochondria for each sample (controls or treated samples) was added to three wells for each treated or vehicle control sample. A total of 2 µL of 10 mM NADH was added per well to begin the reaction. Complex I activity was measured by monitoring the reduction in DCIP at 620 nm using a Dyne MRX micro-plate reader (Dyne technologies, Chantilly, VA, USA). Blank well values (without DCIP) were subtracted from all other measurements. The complex I activity rate was calculated by subtracting the rate as measured in the presence of RO from that obtained in the absence of RO. Complex I activity of treated cells is expressed as a percentage of the vehicle control and assumes that complex I activity of the mitochondria isolated from the vehicle control is 100%. The concentrations of DCIP, RO, and mitochondria were calibrated before the experiments. For complex I enzyme kinetics, NADH was used with final concentrations of 10, 20, 40, 80, and 160 µM.

#### 2.5.4. Mitochondrial Complex III Assay

To measure complex III activity, cellular lysates were prepared following the procedure described in Spinazzi et al. [29]. The protein concentration of cellular samples was measured using a Bradford assay. To For each assay in a 96-well plate, 200 uL of complex III assay buffer, 2 μL of the complex III inhibitor, and antimycin A (1 mg/mL) were added to three wells, respectively. Then, 10 μg of cell lysate from neurotoxicant exposures and vehicle-treated samples were added (3 wells for each sample). The reaction was started by adding 4 μL of 5 mM DUB and pipette mixing. The plates were incubated at 37 °C for 5 min. Blank wells (without cell lysate) were subtracted from all wells. Complex III activity was measured by monitoring the reduction in cytochrome c at 550 nm using a Dyne MRX micro-plate reader (Dyne technologies, Chantilly, VA, USA). The complex III activity rate was calculated by subtracting the rate as measured in the presence of antimycin A from the rate obtained in the absence of antimycin A. For complex III enzyme kinetics assays, cytochrome c was used at final concentrations of 10, 20, 40, 80, and 160 µM. The following equation was used to calculate enzyme activity:

Specific complex I and III enzyme activity (nmol min^−1^ mg^−1^) = (∆ Absorbance/min × 1000)/[(extinction coefficient × volume of sample used in mL) × (sample protein concentration in mg mL^−1^)].

Experiments were conducted at least four times for each concentration of sample. The extinction coefficient was estimated at 6.2 mM^−1^ cm^−1^ for NADH for complex I-specific activities, whereas a coefficient of 18.5 mM^−1^ cm^−1^ for reduced cytochrome c was used for estimation of the specific activities of mitochondrial complex III.

### 2.6. Lactate Production Assay

Lactate is a metabolite produced as a consequence of anaerobic glycolysis, the main compensatory pathway utilized by as a consequence of decreased ATP production in response to mitochondrial complex inhibition. SH-SY5Y cells were seeded in 24-well plates (5 × 10^5^ cells/well) and differentiated into both the DA and CH phenotypes. Undifferentiated SH-SY5Y cells were also seeded in 24-well plates at a density of 5 × 10^4^ cells/well. Cells were treated with PQ, RO, or MPTP at their estimated MTT EC_50_ concentrations (15, 90, and 150 µM, respectively) and at the lower concentration of 10 µM for 24 h. Media were removed, and lactate levels were measured using a commercial lactate assay kit (Biovision, Mountain View, CA, USA) following the manufacturer’s instructions. The assay was conducted in 96-well plates containing 25 µL of media, 25 µL of assay buffer, and 50 µL of the reaction mix in each well. The plates were kept in the dark at room temperature, and then, after 30 min, the absorbance was read at 570 nm wavelength using a Dyne MRX micro-plate reader (Dyne technologies, Chantilly, VA, USA). All assay points were performed in triplicate.

### 2.7. Measurements of Markers of Oxidative Stress

To study the effect of the neurotoxicants on cellular redox status, cells were treated with PQ, RO, and MPTP at their estimated MTT EC_50_s (15, 90, and 150 µM, respectively) and at a lower concentration of 10 µM for 24 h, and oxidative stress biomarkers were assayed.

#### 2.7.1. Reactive Oxygen Species (ROS) Assay

This assay was conducted according to a method described in a previous publication [27]. Briefly, 24 h post-exposure, the media were removed, and each well was washed with phosphate-buffered saline (PBS). Then, 100 μL of dichlorofluorescindiacetate (DCFDA) solution (25 µM in Hank’s solution) was added and incubated in the dark with the cells for 45 min at 37 °C. Wells were washed again, and the absorbance was read in a Dyne MRX micro-plate reader (Dyne technologies, Chantilly, VA, USA) using an excitation wavelength of 485 nm and an emission wavelength of 535 nm. The average of blank readings was subtracted from all readings, and the ROS content of treated samples was represented as a percentage of the vehicle control. Antimycin A (10 µM) was also used as a positive control after a 30 min exposure time. Experiments were conducted in triplicate for each data point.

#### 2.7.2. NF-E2-Related Factor 2 (NRf2)

NRf2 was measured using a commercial kit (ab207223, Abcam, Cambridge, UK) following the manufacturer’s protocol. Briefly, the assay was conducted on a 96-well plate that had an immobilized double-stranded DNA sequence with the Nrf2 consensus binding site. To each well, the following was added: 40 μL of binding buffer and 15 μg of nuclear extract (prepared according to the kit manufacturer’s guidance) in 10 μL of lysis buffer. The plate was incubated on a rocking platform for 1 h at room temperature, and then each well was washed twice, followed by incubation for 1 h with an anti-Nrf2 primary antibody (100 μL/well). The wells were washed, and then secondary antibodies (1/1000 dilution) were added for 1 h at room temperature. An initiating solution was added (100 μL/well), and plates were incubated in the dark for 10 min, then a stopping solution was added. The absorbance was read by a plate reader (Perkin Elmer, Ueberlingen, Germany) at 450 nm. Blank readings were subtracted from all absorbance values. Experiments were performed in triplicate.

#### 2.7.3. Thiobarbituric Acid Reactive Substances (TBARS)

TBARS were quantified as markers of lipid peroxidation [30] according to the method described by Alam et al. [31]. Briefly, to 1 mL of sample solution, 2 mL of 20% (*w*/*v*) trichloroacetic acid and 2 mL of 0.67% (*w*/*v*) thiobarbituric acid were added, and then the mixture was heated in a boiling water bath for 10 min. The sample was cooled and then bench-centrifuged at 3000 rpm for 20 min. The supernatant was removed, the sample was transferred to a microtiter plate, and the absorbance was read at 552 nm using a Dyne MRX micro-plate reader (Dyne Technologies, Chantilly, VA, USA).

#### 2.7.4. Antioxidant Enzyme Activities

The activities of the antioxidant enzymes catalase (CAT) and superoxide dismutase (SOD) were measured. CAT activity was quantified using a commercial kit (Abcam, Cambridge, UK) following the manufacturer’s protocol. This assay is based on the ability of cellular CAT to react with hydrogen peroxide (H_2_O_2_) to produce oxygen and water. The remaining H_2_O_2_ is measured colorimetrically at 570 nm. CAT activities were expressed as µmoles of H_2_O_2_ consumed/min/mg of the sample protein. SOD activity measurements were conducted following the method described by Beauchamp and Fridovich [32]. The assay is based on SOD-mediated inhibition of blue formazan formation by reduction in nitroblue tetrazolium by superoxide anions. The results were normalized to individual sample protein concentrations.

### 2.8. Statistical Analysis

For MTT EC_50_s estimation, the concentration–response relationships were quantified by fitting the data with the following equation:

Y = 100/(1 + ([D]/EC_50_) h)

where Y is the magnitude of the drug effect normalized as a percentage of the control effect, [D] is the concentration of the drug, EC_50_ is the drug concentration that produces a half-maximal effect (50%), and h is an index of the slope. The EC_50_ and slope (h) values are quoted with 95% confidence intervals. All data is presented as the mean ± standard error of the mean (SEM). Neurotoxicant effects on mitochondrial complex enzyme kinetics were evaluated by nonlinear regression analyses. A Michaelis–Menten model was used to determine enzymatic Km, the concentration of substrate at half-maximal enzyme velocity. Three or more groups of data were analyzed using either a one-way or two-way ANOVA with Dunnett’s or Bonferroni post-tests. A Spearman rank-order correlation coefficient was undertaken to consider the association between cell viability measured using MTT and that determined by ATP levels. All statistical analyses were conducted using GraphPad Prism 5 (GraphPad Software Inc., San Diego, CA, USA), and statistical significance was defined as *p* < 0.05.

## 3. Results

### 3.1. The Cytotoxic Effects of Paraquat, Rotenone, and MPTP

**Figure 1:**
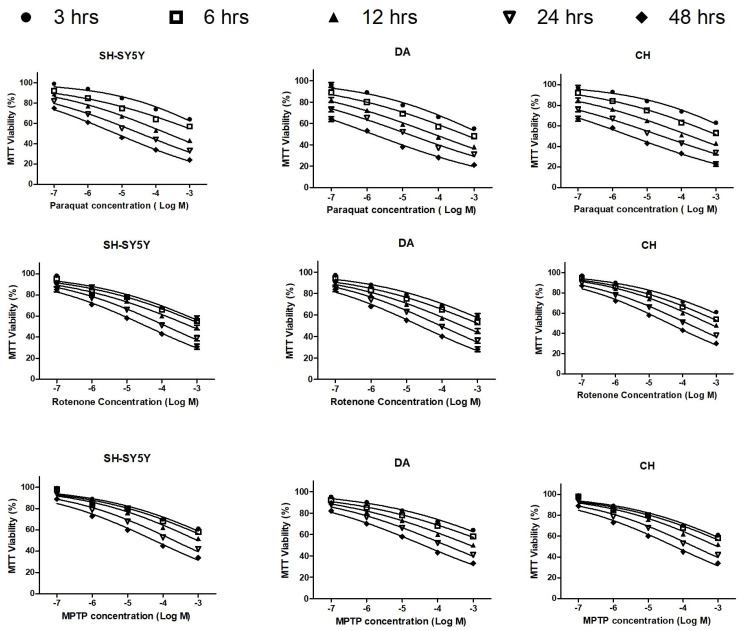
The cytotoxic effect of neurotoxicants on neuroblastoma cells. Paraquat, rotenone, and MPTP were applied to undifferentiated human neuroblastoma cells (SH-SY5Y) or differentiated dopaminergic (DA) or cholinergic (CH) cells at concentrations of 0.1, 1, 10, 100, and 1000 µM for 3, 6, 12, 24, and 48 h, and cellular viability was quantified using an MTT assay. After subtraction of blank well readings, cell viability was expressed as a percentage of the corresponding control level (assumed to be 100%). Data were expressed as means ± SEM. Results were analyzed by a two-way ANOVA with a Bonferroni post-test.

The tested neurotoxicants were significantly cytotoxic to the three studied cell phenotypes in concentration- and exposure duration-dependent patterns, as assessed using an MTT assay (Figure 1 and Supplementary Table S1). The concentration of agents that produced 50% effective concentrations (EC_50_s) was estimated and is included in Table 1. PQ was the most cytotoxic agent to all of the cell phenotypes, with lower estimated EC_50_s at all exposure time points (Table 1). PQ at 1 µM was significantly cytotoxic to differentiated cells even at 6 h post-exposure, but only induced cytotoxicity 12 h post-treatment for undifferentiated cells. Twenty-four hours post-exposure, differentiated neuroblastoma cells were more sensitive to PQ and RO than undifferentiated cells, with the DA cells more vulnerable to neurotoxicity than CH cells (Table 1 and Supplementary Table S1). However, undifferentiated cells were the most sensitive to MPTP treatment (Table 1).

### 3.2. Bioenergetic Analysis of the Effects of Paraquat, Rotenone, and MPTP

**Figure 2:**
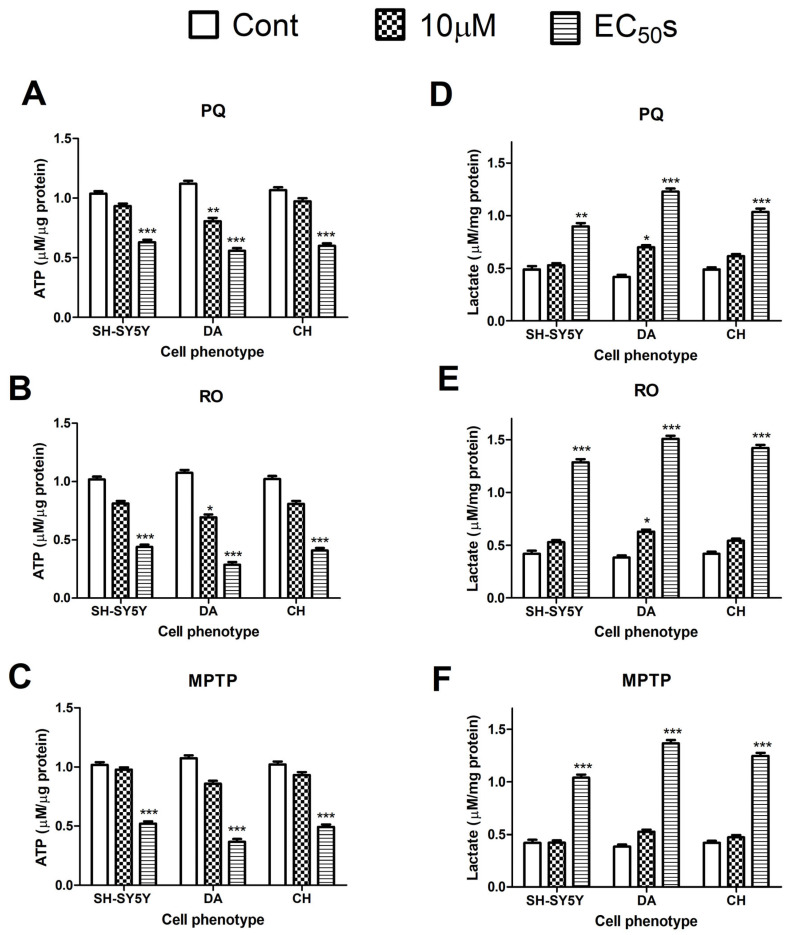
The effects of the neurotoxicants on cellular ATP and lactate levels. Undifferentiated human neuroblastoma cells (SH-SY5Y) or differentiated dopaminergic (DA) or cholinergic (CH) cells were treated with paraquat (PQ), rotenone (RO), or MPTP, and the levels of ATP (**A**–**C**) and lactate (**D**–**F**) were quantified. Neurotoxicants were applied at concentrations of 10 µM as well as at their MTT-estimated EC_50_s (PQ at concentrations of 35, 15, and 25 µM; RO at concentrations of 150, 90, and 120 µM; and MPTP at concentrations of 150, 170, and 250 µM for SH-SY5Y, DA, and CH cells, respectively) for 24 h. After subtraction of the blank readings from all values, readings were generated using standard curves. ATP and lactate levels were normalized to the cellular protein concentrations of the corresponding samples. All experiments were performed in triplicate. Data were expressed as means ± SD. A two-way ANOVA with a Bonferroni post-test was used to compare the different sets of data. For marked significance, * *p* < 0.05, ** *p* < 0.01, *** *p* < 0.001.

All neurotoxicants significantly reduced ATP production in the tested cells when they were exposed to their MTT EC_50_ concentrations (by 40–50% for PQ, 60–73% for RO, and 49–66% for MPTP), broadly consistent with the reduction in cell viability detected using the MTT method. A Spearman rank-order correlation coefficient analysis showed a significant positive correlation value of 0.7950 (*p*-value = 0.0138) for the association between cell viability measured using MTT and that determined by ATP levels (Supplementary Figure S3). After exposure to the neurotoxicants at 10 µM concentrations, only PQ and RO induced a significant decrease in ATP production in DA cells but not CH cells or undifferentiated SH-SY5Y cells (Figure 2A–C). A two-way ANOVA test concluded that the difference in cell line vulnerability to the tested neurotoxicants was agent-specific and related to both concentration and exposure durations. A Bonferroni post-test revealed that DA cells were more vulnerable to the inhibitory effect of the three neurotoxicants with regard to ATP levels (Supplementary Table S2).

All three neurotoxicants significantly increased lactate production when cells were treated with their MTT-estimated EC_50_s. PQ also increased lactate production in DA cells at a lower concentration of 10 µM (Figure 2D–F). Lactate levels in DA cells also displayed the highest increase (370 ± 32%) from control levels in response to the tested neurotoxicants. Significant differences were detected for the neurotoxicant effects when compared between the cell phenotypes regarding lactate production, and there was also a significant difference between the effects of the different agents in the same cell phenotype (Supplementary Table S2).

**Figure 3:**
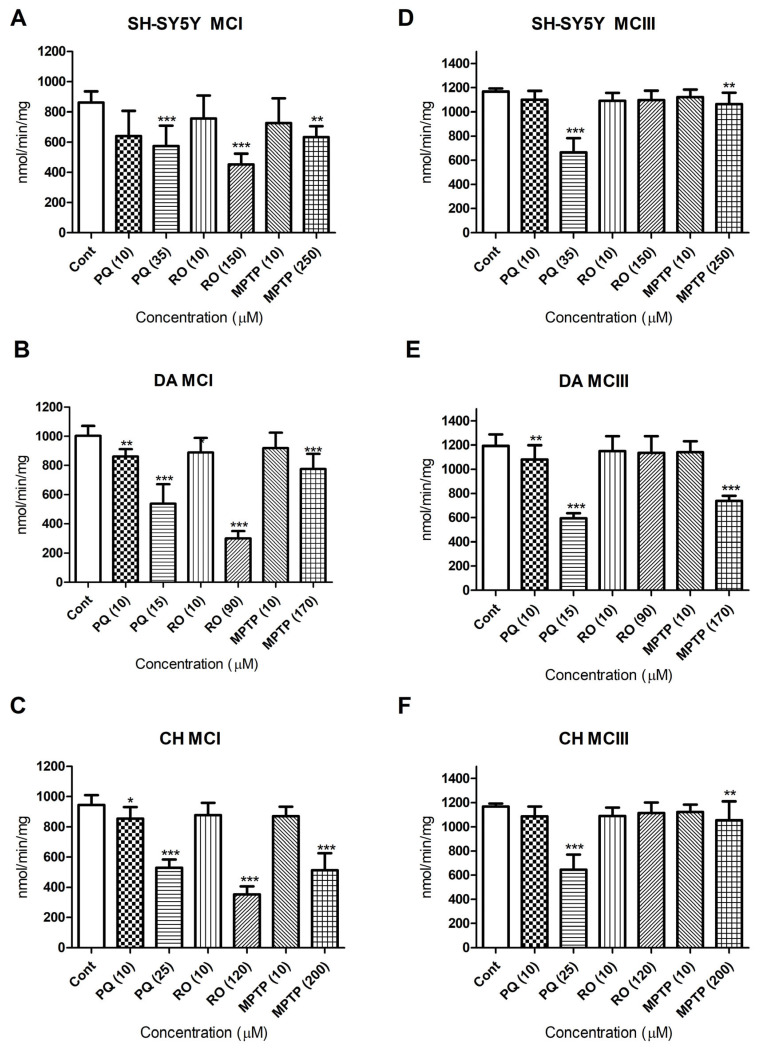
The effect of the neurotoxicants on mitochondrial complex I and complex III activities. Undifferentiated human neuroblastoma cells (SH-SY5Y) or dopaminergic (DA) or cholinergic (CH) cells were incubated with paraquat (PQ), rotenone (RO), or MPTP, and the activity of mitochondrial complex I (**A**–**C**) and mitochondrial complex III (**D**–**F**) was quantified. The neurotoxicants were applied at concentrations of 10 µM as well as at their MTT-estimated EC_50_s (PQ at concentrations of 35, 15, and 25 µM; RO at concentrations of 150, 90, and 120 µM; and MPTP at concentrations of 150, 170, and 250 µM for SH-SY5Y, DA, and CH cells, respectively) for 24 h. Specific complexes I and II enzyme activity were calculated as (nmol min^−1^ mg^−1^) = (∆ Absorbance/min × 1000)/[(extinction coefficient × volume of sample used in mL) × (sample protein concentration in mg mL-L)]. Experiments were conducted at least four times for each concentration sample. The molar extinction coefficient was estimated at 6.2 mM^−1^ cm^−1^ for NADH for use in the measurement of complex I-specific activities, and 18.5 mM^−1^ cm^−1^ was used for reduced cytochrome c for estimation of the specific activities of mitochondrial complex III. Data are presented as means ± SD. A two-way ANOVA with a Bonferroni post-test was used to compare the different sets of data. For marked significance, * *p* < 0.05, ** *p* < 0.01, *** *p* < 0.001.

The effect of PQ, RO, and MPTP on mitochondrial complexes I and III was evaluated. All three tested neurotoxicants significantly reduced mitochondrial complex I activity at their MTT-estimated EC_50_s and in all three cell phenotypes (Figure 3A–C). RO was the most potent inhibitor against complex I and reduced its activity to approximately 54% of the vehicle control cells in undifferentiated SH-SY5Y cells and to 30 and 37% in differentiated DA and CH cells, respectively.

When the neurotoxicants were applied to cells at their MTT EC_50_ concentrations, PQ was the most potent inhibitor against mitochondrial complex III and decreased its activity to approximately 60, 49, and 54% of controls for SH-SY5Y, DA, and CH cells, respectively (Figure 3D–F). By comparison, RO at either 10 µM or at the MTT EC_50_ concentrations did not significantly inhibit complex III in either of the cell phenotypes. Interestingly, the application of PQ at 10 µM significantly inhibited complexes I and III in DA cells as well as complex I in CH cells, whereas RO and MPTP showed non-significant effects on complexes I and III at this concentration. A two-way ANOVA confirmed a difference between the vulnerability of the cell phenotypes to the neurotoxicants with regard to mitochondrial complex inhibition. A Bonferroni post-test showed that DA cells were more sensitive to the inhibitory effects of the three neurotoxicants on mitochondrial complex I activity without a significant difference regarding their effects on mitochondrial complex III activity (Supplementary Table S2).

#### Enzyme Kinetic Effects of Paraquat, Rotenone, and MPTP

Bioenergetics data were further supported by an examination of mitochondrial enzyme kinetics, and these values are summarized in Table 2. For mitochondrial complex I kinetics, the highest Km and lowest Vmax values were in RO-treated samples for the three tested cell phenotypes. In addition, DA cells displayed the highest Km and lowest Vmax in comparison to the CH cells or the undifferentiated SH-SY5Y cells in response to the three tested neurotoxicants (Table 2).

### 3.3. The Effects of Paraquat, Rotenone, and MPTP on Cellular Oxidative Stress

**Figure 4:**
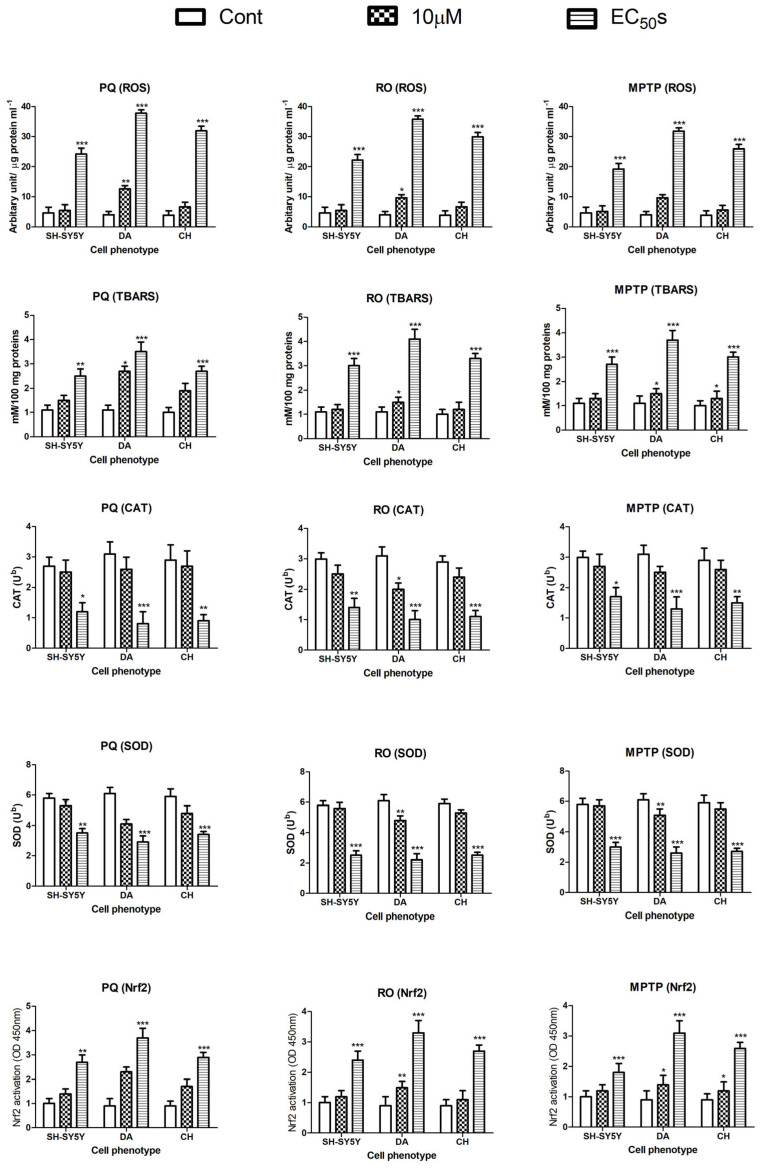
The effect of neurotoxicants on markers of oxidative stress. The levels of reactive oxygen species (ROS), thiobarbituric acid reactive substances (TBARS), catalase (CAT) activities, superoxide dismutase (SOD) activities, and nuclear NF-E2-related factor 2 (NRf2) were quantified in response to neurotoxicant incubations with undifferentiated human neuroblastoma cells (SH-SY5Y) and differentiated dopaminergic (DA) and cholinergic (CH) cells. The neurotoxicants were applied at concentrations of 10 µM as well as their MTT-estimated EC_50_s (PQ at concentrations of 35, 15, and 25 µM; RO at concentrations of 150, 90, and 120 µM; and MPTP at concentrations of 150, 170, and 250 µM for SH-SY5Y, DA, and CH cells, respectively) for 24 h. Data are presented as means ± SD. A two-way ANOVA with a Bonferroni post-test was performed to compare the different sets of data. For marked significance, * *p* < 0.05, ** *p* < 0.01, *** *p* < 0.001.

To assess the effects of the neurotoxicants on markers of oxidative stress, the levels of ROS, lipid peroxidation (TBARS), the activities of the antioxidant enzymes CAT and SOD, and the expression of nuclear Nrf2 were quantified. PQ, RO, and MPTP all increased the levels of ROS, TBARS, and nuclear Nrf2 in all cell phenotypes, and there was a parallel and significant inhibition of CAT and SOD activities (Figure 4). The three neurotoxicants all significantly increased the markers of oxidative stress in the treated cells when applied at their MTT-estimated EC_50_s, while their effects at the lower tested concentration (10 µM) varied between the cell phenotypes, with DA cells notably vulnerable to PQ and RO (Figure 4). A two-way ANOVA confirmed that there were significant differences in the effect of the neurotoxicants between the cell phenotypes for the oxidative stress biomarkers (Supplementary Table S3).

## 4. Discussion

Neurotoxicant models have improved our understanding of the mechanisms associated with environmental chemical exposure and the induction of parkinsonian phenotypes. The three studied neurotoxicants (PQ, RO, and MPTP) have been used by researchers to study neurotoxic effects using isolated mitochondria, cultured cells, and animal models of PD [25,33,34,35,36]. In this study, we provide data that consider a direct comparison of their cytotoxicity, their impact on cellular bioenergetics, and whether this is influenced by cellular differentiation. The current data showed that the neurotoxicants were cytotoxic to the three cell phenotypes that reflected concentration and exposure duration. When applied at concentrations that induced a 50% reduction in cell viability, all neurotoxicants significantly reduced cellular ATP levels and the activities of mitochondrial complex I and III enzymes. There was also a parallel increase in lactate production, although some responses were agent- and cellular phenotype-specific. PQ was especially toxic to DA cells and then CH cells when compared with undifferentiated cells, such that 10 µM was sufficient to significantly decrease ATP production and mitochondrial complex III activity only in DA cells. RO was the most potent inhibitor of mitochondrial complex I but did not inhibit mitochondrial complex III activity, even at a high concentration. All neurotoxicants were inducers of cellular redox stress, measured by the production of ROS, levels of TBARS, and nuclear expression of Nrf2. Furthermore, neurotoxicant exposures resulted in a reduction in the activities of the antioxidant enzymes catalase and superoxide dismutase. At a 10 µM exposure to PQ or RO, oxidative stress biomarkers were significant in DA cells. Collectively, this study underscores the importance of mitochondrial disruption and oxidative stress in PQ, RO, and MPTP-induced cytotoxicity and that neuronal phenotypes display differential vulnerability to these neurotoxicants. These differential effects on cell viability mediated through oxidative stress provide a theoretical basis for brain regional cytotoxic-specific effects, most notably the association between exposure to PQ and the toxicity to DA cells observed in parkinsonian phenotypes.

Experiments were conducted using SH-SY5Y cells, a neuroblastoma cell line. SH-SY5Y cells have been extensively used as a model in neurotoxicology and can be differentiated into DA and CH phenotypes [25,36]. Furthermore, the bioenergetic profile of differentiated SH-SY5Y cells displays hallmarks typical of neurons, including bioenergetic reserve capacity [36], and all three neurotoxicants have been tested using these cells [25,37,38,39]. After differentiation, SH-SY5Y cells have an active dopamine transporter [25,40]. The type and levels of expression of neurotransmitter receptors and transporters, including the dopamine active transporter (DAT), can be influenced by agents that promote differentiation. Primary DA or CH neurons from rodents may provide a closer model of human neuronal counterparts, but these neuronal populations are often heterogeneous (as opposed to a homogeneous cell line) and more difficult to obtain in suitable quantity and will require ethical approval [41]. Hence, the SH-SY5Y cell line can provide a catecholaminergic phenotype since it possesses the machinery to synthesize dopamine and noradrenaline and thereby provides a suitable means to evaluate agent neurotoxicity in vitro and therefore has been extensively used as a model in neurotoxicity and PD research [42,43]. Furthermore, although SH-SY5Y cells display genetic abnormalities due to their cancerous origin, most genes and pathways dysregulated in PD development remain intact [44].

Idiopathic PD patients may have lost approximately 50% of the DA neurons in the nigrostriatal pathway [45]. Hence, for these in vitro studies, concentrations of toxic agents were employed that caused approximately 50% cell death, albeit from acute toxicity rather than the chronic progressive degeneration that typifies PD. The MTT assay indicated that PQ was the most potent toxic agent, which may reflect that PQ has some mechanisms of neurotoxicity distinct from those induced by RO or MPTP and/or may be a more powerful redox stressor [46]. In keeping with our results, other studies have shown that PQ concentrations of 10–25 µM induce a 25–50% loss of SH-SY5Y cell viability [47], and for clinical relevance, the concentration of 10 µM of PQ that was studied is the approximate plasma concentration threshold at 4 h post-exposure for human survival (2 mg/L, approximately 10.7 µM) [48]. Interestingly, the cytotoxicity of all three tested agents was highest in the DA cells compared to either CH or undifferentiated cells, highlighting DA cell vulnerability and the potential for PQ-induction of a parkinsonian phenotype.

Mitochondria are the primary sites for cellular respiration and adenosine triphosphate (ATP) synthesis by oxidative phosphorylation through the Krebs cycle and the passage of electrons along the mitochondrial chain of complexes (mitochondrial complexes I–IV), which release energy required to pump protons into the intermembrane space to generate the proton electrochemical gradient required for ATP synthesis (mitochondrial complex V). Therefore, mitochondrial complexes I and III play a crucial role in this precious chain of complexes, but they are common targets of toxic agents that can inhibit their activities and thereby shut down cellular bioenergetics. In addition, they are a common site for the leakage of protons and the generation of hazardous ROS [49]. Indeed, mitochondrial dysfunction may be one of the major mechanisms for DA degeneration associated with PD [50,51]. In the present study, we show that exposure to PQ, RO, and MPTP can result in a reduction in ATP levels, indicative of mitochondrial dysfunction [52,53,54]. Moreover, we show that all agents inhibited mitochondrial respiratory chain complex I, and this correlated with the induction of ROS and cell death. Mitochondrial complex III inhibition was also observed for PQ and MPTP treatments, but not significantly for RO. Furthermore, neurotoxicants induce cellular ROS production in a concentration-dependent manner. An inhibition of complex I and associated impairment of mitochondrial respiration leads to oxidative damage to proteins, lipids, and DNA by ROS. Ultimately, excessive ROS and cellular damage will trigger the activation of mitochondrial-dependent apoptotic machinery and the induction of cell death [55]. Hence, this is presumably the primary mechanism of the cytotoxicity induced by the three neurotoxicants.

PQ is a weak inhibitor of mitochondrial complex I but is still able to trigger the production of ROS and oxidative damage. However, its toxicity to DA neurons can be imposed by a mechanism that is independent of complex I inhibition and the DAT [46]. Instead, PQ accumulates in mitochondria and acts as a potent redox cycler that converts free radicals interacting with molecular oxygen to form superoxide and other ROS [12,13]. In addition, PQ can elevate ROS generation through glutamate excitotoxicity by Ca^2+^ efflux, depolarization of N-methyl-D-aspartate (NMDA) receptor channels, and activation of non-NMDA receptor channels, although activation of NMDA receptors may be limited in SH-SY5Y cells [56]. Therefore, our research study outcomes are consistent with previous studies suggesting that PQ can induce oxidative stress through mechanisms that in part involve ROS generation and inhibition of mitochondrial complex I, but this may be augmented by additional mechanisms that were not studied further.

RO, a more specific mitochondrial complex I inhibitor, also damages DA neurons through the generation of ROS, resulting in oxidative stress [21]. Other mechanisms, such as the NMDA receptor-mediated bioenergetic crisis due to depleted ATP levels and cell death due to mitochondrial depolarization that arises from the opening of the mitochondrial permeability transition pore, may contribute to RO toxicity [21].

Treatment of cells with MPTP revealed that it was a relatively weak inhibitor of mitochondrial complexes I and III, although sufficient to induce ROS and cellular redox stress. However, in addition to mitochondrial complex effects, MPTP can also induce direct changes in the mitochondrial proteome [57,58]. Surprisingly, MPTP was more cytotoxic to undifferentiated cells than either PQ or RO (Table 1). This could reflect that differentiation with retinoic acid increases the expression of the *mdrl* (multidrug resistance-1)/P-glycoprotein (P-gp) gene [59]. P-gp is a transmembranous protein transporter involved in detoxification by ATPase-driven cytotoxic drug efflux [60]. Hence, undifferentiated cells could accumulate more MPTP within cells due to the lack of RA-induced expression of P-gp and the associated reduction in MPTP efflux, but we have not investigated this further.

Although we have considered the toxicity of MPTP, it is likely that its metabolite, MPP^+^, will be a more potent toxicant to the DA cells, assuming active update by dopamine transporters [21]. However, MPTP is the parent (lipid-soluble) compound that is often used as the chemical means to induce Parkinsonian phenotypes in experimental models of PD [33,34,35] and may also be notably toxic. MPP^+^ was approximately 100-fold more cytotoxic than MPTP to PC12 cells [61], but the conversion of MPTP to MPP^+^, at least by primary astrocytes, was only approximately 20% complete after 24 h [62]. Hence, under acute conditions, cytotoxicity to undifferentiated and differentiated neuroblastoma cells from MPTP may be expected at concentrations of ≥1 µM (Figure 1).

The three studied neurotoxicants, PQ, RO, and MPTP, were cytotoxic to undifferentiated and differentiated cells, but their phenotypic toxicity was not equitable. Notably, DA cells, followed by the CH phenotype, were the most vulnerable to neurotoxicant damage and loss of cell viability. All of the neurotoxic agents significantly decreased ATP levels, and the decline in ATP levels was similar to that induced by MTT and thereby provided an alternative means to validate the neurotoxicant-induced decline in cell viability, as observed in other studies [24,55]. The neurotoxicants inhibited either mitochondrial complex I or complex III activity or both, with a parallel increase in cellular lactate production. Likewise, all neurotoxicants induced the production of ROS and triggered cellular redox stress. However, data from bioenergetics and oxidative stress analyses confirmed that the DA cells were the most susceptible to mitochondrial disruption, induction of oxidative damage, and therefore cytotoxicity. Our previous studies have reported that there are brain regions more vulnerable to the cytotoxic effects of certain pesticides [63] and that at a cellular level, differentiation renders SH-SY5Y cells more vulnerable to neurotoxicity from organophosphate and carbamate pesticides [24]. The process of differentiation results in a change in the proteome with the expression of a number of mature neuronal markers, including cytoskeletal proteins such as βIII-tubulin and microtubule-associated proteins (MAP-2 and MAP-tau) required for the production of neuritic projections. Some of these proteins may be oxidatively damaged in response to neurotoxicants and contribute to their acute or chronic toxicity [24]. Hence, at present, we can only speculate on the rationale for the differential toxicity of PQ, RO, and MPTP and the relative vulnerability of DA cells, but this could arise from the altered expression of proteins vulnerable to redox damage.

## Figures and Tables

**Table 1:** Estimated EC_50_ concentrations for the cytotoxic effects of paraquat (PQ), rotenone (RO), and MPTP. Undifferentiated human neuroblastoma cells (SH-SY5Y) and differentiated dopaminergic (DA) and cholinergic (CH) cells were treated with neurotoxicants at concentrations of 0.1, 1, 10, 100, and 1000 µM for 3, 6, 12, 24, and 48 h, and the estimated EC_50_ concentrations were calculated. Data are shown as means in µM with a 95% confidence interval (CI) range in brackets.

	3 h	6 h	12 h	24 h	48 h
PQ					
SH-SY5Y	5676 (3878–8306)	2463 (1735–3494)	222.3 (183.1–270)	35.5 (30.4–41.4)	6.7 (5.9–7.5)
DA	1612 (1228–2117)	495.7 (399.3–615.5)	72.91 (62.5–85.1)	13.9 (11.9–16.4)	1.4 (1.2–1.6)
CH	5804 (4243–7938)	1325 (1044–1682)	187.3 (156.1–224.7)	27.21 (23.4–31.6)	3.337 (2.9–3.8)
RO					
SH-SY5Y	2668 (1789–3979)	1982 (1423–2760)	706.4 (551.1–905.5)	150.7 (119.3–165.9)	38 (33.4–43.3)
DA	3291 (2278–4753)	1441 (1083–1917)	359.4 (289.4–446.2)	90.3 (75.2–108.3)	23.4 (20.4–26.8)
CH	4524 (3157–6483)	1590 (1246–2029)	586.8 (479.1–718.8)	123 (115–153.9)	38.2 (33.4–43.7)
MPTP					
SH-SY5Y	2668 (1789–3979)	1982 (1423–2760)	706.4 (551.1–905.5)	150.7 (119.3–165.9)	38 (33.4–43.3)
DA	9244 (6127–13,950)	3961 (2944–5330)	811.1 (649.2–1013)	171.6 (144.6–203.7)	39.3 (34.4–44.9)
CH	9420 (5713–15,530)	3231 (2314–4511)	1143 (867.9–1506)	248.2 (204.9–300.7)	66.2 (57.6–76)

**Table 2:** The effects of neurotoxicants on mitochondrial complex enzyme kinetics. Neurotoxicant effects on mitochondrial complex activities were assessed through a nonlinear regression analysis using a Michaelis–Menten model to determine the enzymatic Km. For kinetic assays, undifferentiated human neuroblastoma cells (SH-SY5Y) and differentiated dopaminergic (DA) and cholinergic (CH) cells were treated with the neurotoxicants paraquat (PQ), rotenone (RO), and MPTP at concentrations of 10 µM as well as their MTT-estimated EC50s: (PQ at concentrations of 35, 15, and 25 µM; RO at concentrations of 150, 90, and 120 µM; and MPTP at concentrations of 150, 170, and 250 µM for SH-SY5Y, DA, and CH cells, respectively) for 24 h. Then, NADH or cytochrome c were used as substrates for enzyme kinetic assays of mitochondrial complex I and complex III, respectively, with final concentrations of 10, 20, 40, 80, and 160 µM. Data were represented as means (in µM) ± SD.

		PQ			RO			MPTP		
Cell Phenotype		SH-SY5Y	DA	CH	SH-SY5Y	DA	CH	SH-SY5Y	DA	CH
MCI										
Control	Vmax	0.5 ± 0	0.5 ± 0	0.5 ± 0	0.5 ± 0	0.5 ± 0	0.5 ± 0	0.5 ± 0.	0.5 ± 0	0.5 ± 0
	Km	38.4 ± 4.9	38.4 ± 4.9	40 ± 5.4	23.4 ± 4.9	38.4 ± 4.9	32.0 ± 5.4	38.4 ± 4.9	38.4 ± 4.9	40.0 ± 5.4
10 µM	Vmax	0.4 ± 0	0.3 ± 0	0.4 ± 0	0.4 ± 0	0.4 ± 0.1	0.4 ± 0	0.5 ± 0	0.4 ± 0	0.5 ± 0
	Km	49.7 ± 2.3	79.4 ± 2	51.4 ± 3.9	38.7 ± 5.5	95.8 ± 14.5	45.7 ± 12.4	40.8 ± 1.1	63.3 ± 12.4	45.3 ± 8.6
EC_50_s	Vmax	0.2 ± 0	0.1 ± 0	0.3 ± 0	0.2 ± 0	0.1 ± 0	0.17 ± 0.01	0.3 ± 0	0.2 ± 0	0.3 ± 0
	Km	113.4 ± 4.4	130.7 ± 12.8	123 ± 10.5	106.5 ± 7.9	179.2 ± 18.9	159.2 ± 15.5	97.3 ± 16.9	114.8 ± 17.5	95.6 ± 11.8
MCIII										
Control	Vmax	0.5 ± 0	0.5 ± 0	0.5 ± 0	0.5 ± 0	0.5 ± 0	0.5 ± 0	0.5 ± 0	0.5 ± 0	0.5 ± 0
	Km	30.9 ± 1.7	29.4 ± 1.3	32.9 ± 2.4	29.1 ± 1.4	33.8 ± 1.4	32.9 ± 2.4	34.3 ± 1.9	29.1 ± 1.4	32.9 ± 2.4
10 µM	Vmax	0.4 ± 0	0.4 ± 0	0.5 ± 0	0.5 ± 0	0.4 ± 0	0.5 ± 0	0.4 ± 0	0.4 ± 0.1	0.5 ± 0
	Km	44.1 ± 3.1	54.9 ± 6.3	52.3 ± 4.8	35.2 ± 1.5	45.9 ± 2.8	39.8 ± 3.3	37.2 ± 1.1	70.6 ± 15.8	40.1 ± 2.9
EC_50_s	Vmax	0.3 ± 0	0.3 ± 0.1	0.3 ± 0.1	0.5 ± 0	0.4 ± 0	0.4 ± 0.1	0.2 ± 0	0.2 ± 0	0.3 ± 0.1
	Km	73.9 ± 13.2	81.6 ± 18.4	92.8 ± 19.2	41 ± 5.5	52.8 ± 9.2	43.7 ± 6.1	92.9 ± 21	115.4 ± 23.2	99.6 ± 23.4

## Data Availability

Data is available from Mansoura University and the University of Nottingham after contact with the corresponding authors Ekramy Elmorsy (ekramyelmorsy@mans.edu.eg) and Wayne Carter (wayne.carter@nottingham.ac.uk).

## Supplementary Materials

The following supporting information can be downloaded at: https://www.mdpi.com/article/10.3390/brainsci13121717/s1, Figure S1: Comparison of undifferentiated and differentiated SH-SY5Y cells using microscopy and Western blotting; Figure S2: MTT assay optimization for SH-SY5Y cells; Figure S3: Spearman’s correlation of cell viability measurements using a MTT method compared with an ATP method; Table S1: Comparisons between the cytotoxic effects of paraquat (PQ), rotenone (RO), and MPTP on the different cell phenotypes; Table S2: Comparisons between the effects on cellular bioenergetics for paraquat (PQ), rotenone (RO), and MPTP on the different cell phenotypes; Table S3: Comparisons between the effects on markers of oxidative stress for paraquat (PQ), rotenone (RO), and MPTP on the different cell phenotypes.
