# Supplementary material for: Differential Effects of Paraquat, Rotenone, and MPTP on Cellular Bioenergetics of Undifferentiated and Differentiated Human Neuroblastoma Cells
Source: Brain Sci. 2023 Dec 14;13(12):1717. doi: 10.3390/brainsci13121717 (PMC10741680; doi:10.3390/brainsci13121717)
Supplement: Supplementary file 1 [file brainsci-13-01717-s001.zip › brainsci-2694927-supplementary.pdf]

brain sciences

MDPI

Supplementary Data

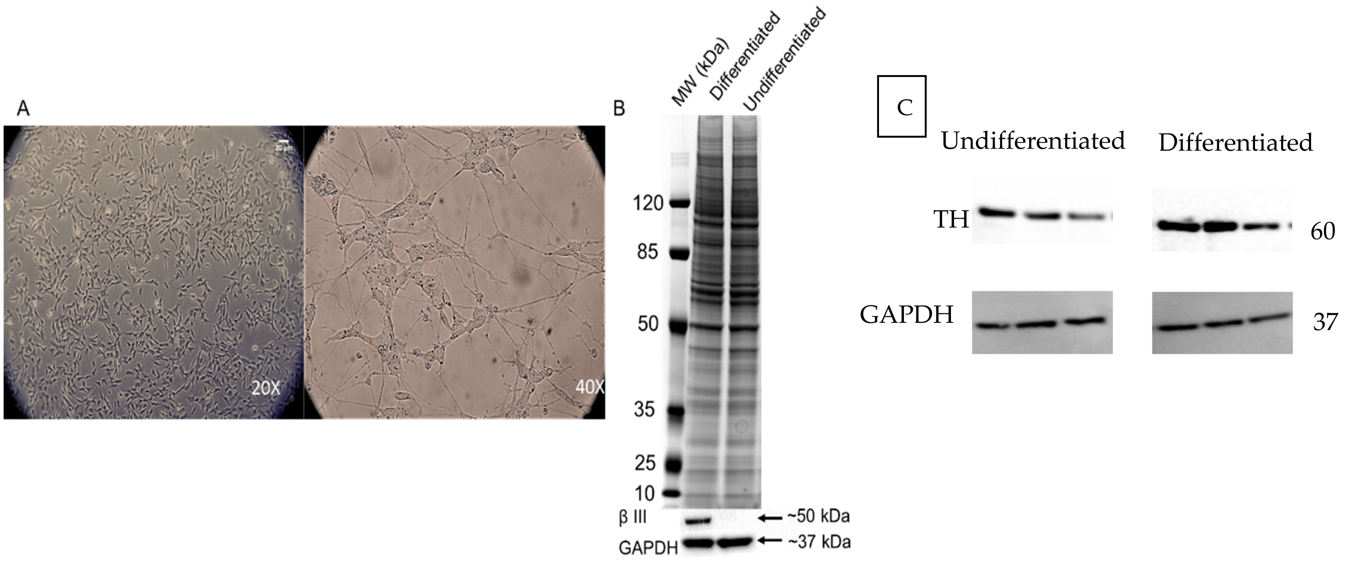

Figure S1. Comparison of undifferentiated and differentiated SH-SY5Y cells using microscopy and Western blotting. **(A)** Light microscopy of undifferentiated SH-SY5Y cells (left panel) or differentiated cells (right panel). Differentiated cells display cell body elongation and dendritic arborization (right panel). **(B)** Differentiated cells express neuronal markers including β-III tubulin. **(C)** Differentiated cells express increased levels of tyrosine hydroxylase (TH). GAPDH, Glyceraldehyde 3-phosphate dehydrogenase.

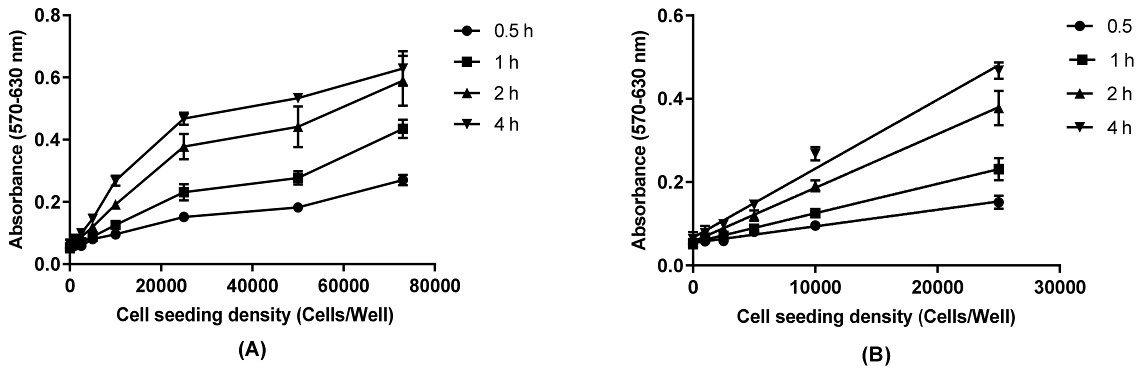

Figure S2. MTT assay optimization for SH-SY5Y cells. (**A**) MTT optimization to determine a linear correlation between the optical density signal and cell seeding density for SH-SY5Y cells after 0.5, 1, 2 and 4 hours. (**B**) Correlation of optical density readings with cell seeding numbers for SH-SY5Y cells.

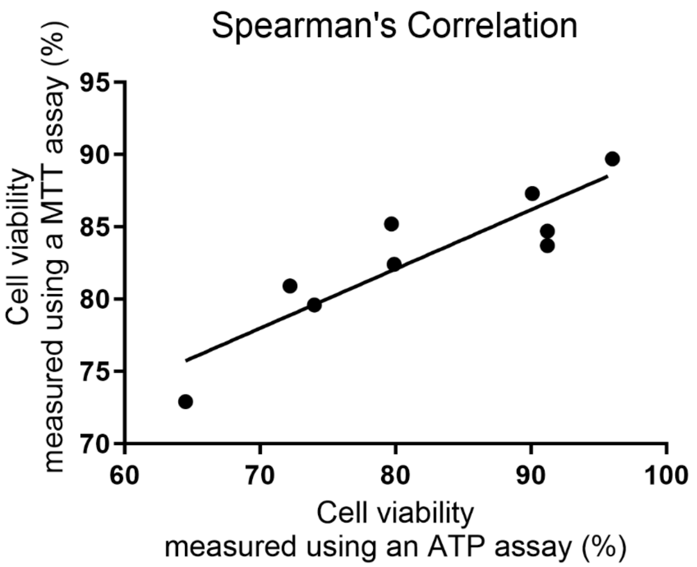

Figure S3. Spearman's correlation of cell viability measurements using a MTT method compared with an ATP method. Undifferentiated SH-SY5Y cells or cells differentiated to a dopaminergic or cholinergic phenotype were treated with 10 μM paraquat, rotenone, or MPTP and the reduction of cell viability (from 100%) determined using a MTT assay or an ATP assay. A Spearman's rank correlation showed a positive (0.7950) and significant (p = 0.0138) correlation for the different cell viability methods.

Table S1. Comparisons between the cytotoxic effects of paraquat (PQ), rotenone (RO), and MPTP on the different cell phenotypes. The effect of cell phenotype and the pesticide concentration on cell viability 24 hours after exposure as measured using a MTT assay was considered using a two-way ANOVA for undifferentiated human neuroblastoma cells (SH-SY5Y) and differentiated dopaminergic (DA) and cholinergic (CH) cells. For marked significance, *$p < 0.05$, **$p < 0.01$, ***$p < 0.001$, and ns refers to non-significant effects.

	**PQ**	**RO**	**MPTP**	**PQ**	**RO**	**MPTP**	**PQ**	**RO**	**MPTP**
Interaction	***	***	*						
Cell phenotype	***	***	***						
Conc	***	***	***						
	DA vs CH			DA vs SH-SY5Y			CH vs SH-SY5Y		
Control	ns	ns	ns	ns	ns	ns	ns	ns	ns
−7.0	**	***	***	***	ns	**	***	***	ns
−6.0	ns	***	***	**	**	ns	ns	***	ns
−5.0	ns	***	**	***	**	ns	ns	***	ns
−4.0	***	***	ns	ns	ns	ns	ns	***	ns
−3.0	**	***	ns	***	**	***	ns	***	ns

Table S2. Comparisons between the effects on cellular bioenergetics for paraquat (PQ), rotenone (RO), and MPTP on the different cell phenotypes. Undifferentiated SH-SY5Y cells or cells differentiated to a dopaminergic (DA) or cholinergic (CH) phenotype were treated with paraquat (PQ), rotenone (RO), or MPTP for 24 hours and the effects on ATP levels, lactate production and mitochondrial complex enzyme activities of neurotoxicant concentration on each cell phenotype evaluated using a two way ANOVA.

Assay	Source of variation	% of total variation	*P* value
PQ ATP	Interaction	4.42	<0.0001
	Conc	93.34	<0.0001
	Phenotype	1.10	<0.0001
RO ATP	Interaction	2.20	<0.0001
	Conc	95.83	<0.0001
	Phenotype	1.35	<0.0001
MPTP ATP	Interaction	2.42	<0.0001
	Conc	95.46	<0.0001
	Phenotype	1.39	<0.0001
PQ lactate	Interaction	6.53	<0.0001
	Conc	87.86	<0.0001
	Phenotype	4.83	<0.0001
RO lactate	Interaction	1.04	<0.0001
	Conc	97.88	<0.0001
	Phenotype	0.80	<0.0001
MPTP lactate	Interaction	2.69	<0.0001
	Conc	94.82	<0.0001
	Phenotype	2.10	<0.0001
PQ MCI	Interaction	6.51	<0.0001
	Conc	64.85	<0.0001
	Phenotype	5.32	<0.0001
RO MCI	Interaction	4.56	<0.0001
	Conc	85.73	<0.0001
	Phenotype	0.42	0.0940
MPTP MCI	Interaction	8.29	<0.0001
	Conc	48.65	<0.0001
	Phenotype	14.75	<0.0001
PQ MCIII	Interaction	0.44	0.2618
	Conc	88.34	<0.0001
	Phenotype	0.13	0.4664
RO MCIII	Interaction	0.77	0.8717
	Conc	11.00	0.0003
	Phenotype	4.07	0.0413
MPTP MCIII	Interaction	24.76	<0.0001
	Conc	39.73	<0.0001
	Phenotype	8.18	<0.0001

Table S3. Comparisons between the effects on markers of oxidative stress for paraquat (PQ), rotenone (RO), and MPTP on the different cell phenotypes. Undifferentiated SH-SY5Y cells or cells differentiated to a dopaminergic (DA) or cholinergic (CH) phenotype were treated with paraquat (PQ), rotenone (RO), or MPTP for 24 hours and the effects on markers of oxidative stress of neurotoxicant concentration on each cell phenotype evaluated using a two way ANOVA.

Assay	Source of variation	% of total variation	*P* value
PQ ROS	Interaction	3.81	<0.0001
	Conc	90.07	<0.0001
	Phenotype	4.80	<0.0001
RO ROS	Interaction	4.30	<0.0001
	Conc	90.27	<0.0001
	Phenotype	3.92	<0.0001
MPTP ROS	Interaction	4.94	<0.0001
	Conc	88.12	<0.0001
	Phenotype	4.91	<0.0001
PQ TBARS	Interaction	6.21	<0.0001
	Conc	73.40	<0.0001
	Phenotype	12.90	<0.0001
RO TBARS	Interaction	2.82	<0.0001
	Conc	89.53	<0.0001
	Phenotype	3.26	<0.0001
MPTP TBARS	Interaction	3.31	<0.0001
	Conc	86.98	<0.0001
	Phenotype	3.20	<0.0001
PQ CAT	Interaction	2.43	0.4763
	Conc	85.59	<0.0001
	Phenotype	0.03	0.9782
RO CAT	Interaction	2.33	0.0024
	Conc	86.68	<0.0001
	Phenotype	1.84	0.0014
MPTP CAT	Interaction	1.62	0.1579
	Conc	80.21	<0.0001
	Phenotype	1.03	0.1213
PQ SOD	Interaction	4.71	0.0421
	Conc	85.33	<0.0001
	Phenotype	3.10	0.0348
RO SOD	Interaction	1.43	<0.0001
	Conc	94.38	<0.0001
	Phenotype	0.52	0.0086
MPTP SOD	Interaction	1.26	0.0100
	Conc	92.00	<0.0001
	Phenotype	0.44	0.0904
PQ NrF2	Interaction	4.50	<0.0001
	Conc	82.19	<0.0001
	Phenotype	6.86	<0.0001
RO NrF2	Interaction	3.80	<0.0001
	Conc	84.71	<0.0001
	Phenotype	3.51	<0.0001
MPTP NrF2	Interaction	10.15	<0.0001
	Conc	73.18	<0.0001
	Phenotype	5.85	<0.0001
